# Synthesis, Characterization, and DFT of Some Thiazole Derivatives as Carbon Steel Corrosion Inhibitors in 1 M HCl

**DOI:** 10.1002/cbdv.71633

**Published:** 2026-09-10

**Authors:** Ahmed O. Mahmoud, Ghada Y. Elewady, Ahmed E. Suliman, Abdelfattah M. Ouf, Ghada E. Abdel‐Ghani

**Affiliations:** ^1^ Department of Chemistry Faculty of Science Mansoura University Mansoura Egypt; ^2^ Chemistry Department Faculty of Science Menoufia University Shibin El Kom Egypt

**Keywords:** corrosion inhibition carbon steel in 1 M HCl, DFT, SEM, thiazole derivatives

## Abstract

By reacting 2‐(1‐(4‐aminophenyl)ethylidene)hydrazine‐1‐carbothioamide **(1)** with various α‐halocarbonyl compounds, such as 3‐chloropentane‐2,4‐dione **2** and 1‐chloro‐1‐((4‐substituted phenyl)diazenyl)propan‐2‐one **4a–c,** this work produces a new series of thiazole derivatives **3** and **5a–c**. The new substituted thiazole derivatives **3, 5a, 5b,** and **5c**, were determined and examined for their ability to suppress mild steel corrosion in 1 M HCl using spectroscopic analyses. The findings showed that inhibition efficiency rose with concentration, peaking at 5 g/L (93%). The charge transfer resistance (*R*_ct_) increased from 36 Ω cm^2^ (blank) to 370.87 Ω cm^2^at 5 × 10^−3^ M concentration. It was found to have an efficiency IE% in the order of **5c** 95.10% > **5b** 92.56% > **5a** 89.01% > **3** 83.41% at elevated temperatures (298 K for 5 × 10^−3^ M concentration). While adsorption followed the Langmuir isotherm, itindicates the negative values of Δ*G*ads which indicate a spontaneous adsorption process. The surface analysis results from SEM were consistent with previous experimental results. Moreover, compound **5c**'s energy gap (Δ*E*) = 1.99 eV and a larger value of E_HOMO_, indicating substantial inhibitory activity. The most potent inhibitor was identified by DFT thiazole derivative **5c**. These computations showed that a high corrosion prevention efficiency is a result of the thiazole derivative with group of high electron density.

## Graphical abstract

By reacting 2‐(1‐(4‐aminophenyl)ethylidene)hydrazine‐1‐carbothioamide **(1)** with various α‐halocarbonyl compounds, such as 3‐chloropentane‐2,4‐dione 2 and 1‐chloro‐1‐((4‐substituted phenyl)diazenyl)propan‐2‐one **4a–c,** this work produces a new series of thiazole derivatives **3** and **5a–c**. The new thiazole derivatives **3, 5a, 5b,** and **5c** were determined and examined for their ability to suppress mild steel corrosion in 1 M HCl using spectroscopic analyses. It was found that **5c** has a high efficiency IE% of 95.10%. While adsorption indicates the negative values of Δ*G*ads are spontaneous adsorption processes.

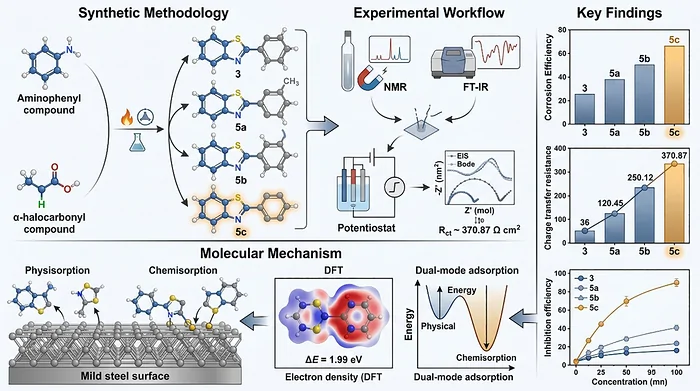

## 1 Introduction

Corrosion is effectively inhibited by organic molecules with many bonds that are adsorption sites, such as heteroatoms of phosphorus, sulfur, nitrogen, and oxygen with a high electron density [1]. A common alloy in many industrial sectors, including chemical processing, petrochemical production, refining, and metal processing equipment, carbon steel is inexpensive, easy to machine, and has special mechanical qualities that set it apart from other metallic alloys [2, 3]. Acid solutions are frequently used in chemical treatment procedures like acid descaling, acid cleaning, and oil‐well acidizing. Carbon steel was selected as the substrate because of its extensive use in industrial installations, pipelines, storage tanks, and acid‐processing equipment. Hydrochloric acid solutions are frequently employed in industrial cleaning, pickling, and acidizing operations, where severe corrosion of carbon steel is often encountered. Moreover, evaluating the corrosion attitude of carbon steel in 1 M HCl under standard laboratory conditions (298 K) provides both industrial relevance and direct comparability with previously reported corrosion inhibition studies. Using corrosion inhibitors is a legitimate strategy to stop corrosion because they lower rates to the necessary level while having no effect on the environment [4, 5, 6]. In an acidic medium, surfactants were more effective at preventing the breakdown of steel when their hydrophilic head groups and hydrophobic tails were doubled [7, 8, 9]. Higher inhibitor activity is seen in the cyclic structure of aromatic organic compounds that contain heteroatoms (oxygen, nitrogen, sulfur, and phosphorus) with a conjugate bond frequently exhibit strong anticorrosion qualities. Inhibitory actions are caused by the electrons and dissociated electron pairs in their chemical structure. This is because they can attach to the metal surface using π‐electrons or lone pairs of electrons [10, 11]. Generally speaking, molecules with both nitrogen and sulfur, like thiazoles, have shown more efficacy in preventing corrosion than those with just one of these elements [12]. Phenyl‐azo of 1,3‐thiazoles‐based derivatives have therefore been created and their effectiveness as corrosion inhibitors investigated. 1,3‐Thiazoles and its derivatives are well known for having relatively minimal toxicity because of their rings that contain nitrogen and sulfur atoms [13, 14, 15]. Along with their derivatives, thiazoles demonstrate a variety of biological actions, such as anticancer [16], antibacterial [17], antioxidant [18], antiviral [19], and cardiovascular effects [20]. They are also significant heterocyclic scaffolds in the drug development process [21]. Thiazole‐based compounds have proven to be particularly efficient corrosion inhibitors in acidic environments. Both nitrogen and sulfur heteroatoms are included into the aromatic thiazole ring, which offers several active sites for adsorption on the steel surface. These heteroatoms' lone‐pair electrons and the thiazole ring's π‐electron cloud allow for robust coordination with the metal's d‐orbitals. This produces a strong, sticky barrier that successfully fends off hostile ions like chloride, preventing additional corrosive damage. By increasing the electron density available for metal coordination, structural changes like adding more heteroatoms or electron‐donating substituents on the thiazole framework might further improve inhibitory efficiency [4, 22, 23, 24, 25]. Therefore, the goal of the current study was to synthesize some new heterocyclic compounds with a 1,3‐ thiazole moiety to repurposing expired it as a corrosion inhibitor for carbon steel in 1 M HCl medium using powerful computational methods to predict the electronic properties of the target molecules. Open potential circuit (OCP), potentiodynamic polarization (PDP), electrochemical impedance (EIS), scanning electron microscope (SEM), Fourier transform infrared microscope (FTIR) and computational density functional theory (DFT) were generated to identify the regions of electrophilic and nucleophilic reactivity

## 2 Result and Discussion

### 2.1 Chemistry

**SCHEME 1:**
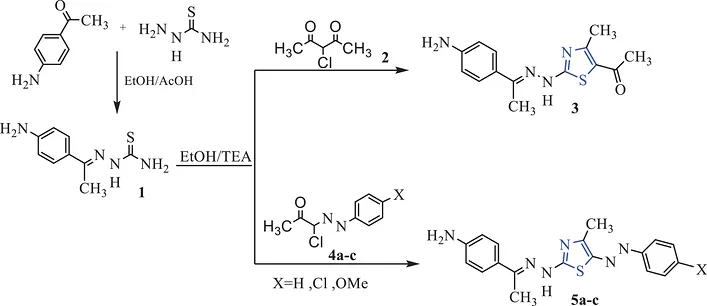
Synthesis of the corresponding 5‐acetyl‐thiazole derivative **3** and 4‐methyl‐5‐(aryl diazenyl)thiazole derivatives **5a–c**.

According to a previously reported method, 2‐(1‐(4‐aminophenyl)ethylidene)hydrazine‐1‐carbothioamide **(1)** was produced in good yields by via the catalytic condensation of 4‐aminoacetophenone with thiosemicarbazide in ethanol using acetic acid [26]. According to Hantzsch reaction, which produces distinct α‐halocarbonyl compounds, the necessary precursor thiosemicarbazone **1** was used in this study to synthesize the target thiazoline compounds. In refluxing ethanol and triethylamine, accomplished the nucleophilic substitution of thiosemicarbazone **1** on chloroacetone **2** provided the conforming intermediate, which produced the matching 5‐acetyl‐thiazole derivative **3** by influencing the intermolecular elimination of water. (Scheme 1) Similarly, 1‐chloro‐1‐(aryldiazenyl)propan‐2‐one **4a–c** in ethanol produced the equivalent 3‐methyl thiazole **5a–c,** which was the result of the base‐catalyzed hydration and cyclization of thiosemicarbazone **(1)** with hydrazonoyl halide (Scheme 1). In order to create S‐alkylated intermediates, the reaction began with S‐alkylation and proceeded to remove hydrogen chloride under the conditions utilized. After that, intermolecular Michael type addition was applied, and by removing water, the procedure created a cyclic adduct, which resulted in the same end product **5a–c**. The suggested compounds' structures were compatible with the spectroscopic analysis as IR, MS, ^1^HNMR, and ^13^CNMR in (Table 1), (Figures S1–S10). Stretching bands for two NH and CO groups may be seen in the IR spectra of structure **3** at *v* = 3182, 3054, and 1732 cm^−1^, respectively. Additionally, its ^1^H NMR spectra revealed singles indications for two methyl and one acetyl proton at *δ* = 2.32, 2.42, and 3.69 ppm. Additionally, NH_2_ and NH (_D2O exchanged)_, groups confirmed by the appearance of singlet signals at 5.5 and 11.4 ppm. The mass spectrum of compound **3** displayed the molecular ion peak at *m/z*: 350.44 (M^+^, 33%) corresponding to the molecular formula C_18_H_18_N_6_S. Additionally, the IR spectra of 4‐methylthiazole compounds **5a–c** demonstrated stretching bands at *ν* = 3300–3339 for (NH, NH_2_) and 1584–1676 cm^−1^ due to N═C of hydrazo form confirming its synthesis. For instance, compound **5a**'s ^1^H‐NMR spectra revealed a novel singlet signal for methyl protons at *δ* 2.43 and 2.51 ppm, as well as signals for the NH_2_ and NH proton and at *δ* 5.79 and 10.52 ppm. Its ^13^C NMR spectra showed a signal at *δ* 15.17 and 16.50 ppm, which is associated with two CH_3_ groups and also signal at *δ* 169.35, 177.14 ppm for (C─NH_2_), and (C═N), respectively. Moreover, Compound **5b**'s ^1^H NMR spectra revealed methyl protons at 2.19 and 2.51 ppm. Additionally, there is an NH_2_ and NH group singlet signals at *δ* 4.19 and 10.88 ppm, respectively. Additionally, ^1^H NMR spectra of compound **5c** revealed novel singlet signal for the CH_3_, at *δ* = 3.72, ppm. And also presence of singlet signal of NH_2_ and NH group at *δ* 4.18 and 10.87 ppm, respectively Moreover, the signals for CH_3_ and C═N at 55.75and 192.89 ppm in the ^13^C NMR spectrum, respectively.

**TABLE 1:** Characterization of synthesized thiazole derivatives **3, 5a, 5b,** and **5c**.

Compounds	IR (KBr)*ν*/cm^−1^	^1^HNMR (400 MHz, DMSO‐*d*_6_) *δ* (ppm)	^13^C NMR (δC/ppm):
*1‐(2‐(2‐(1‐(4‐aminophenyl)ethylidene)hydrazineyl)‐4‐methylthiazol‐5‐yl)ethan‐1‐one (3)*.	3182, 3054 (NH, NH_2_), 1732(CO), 1619(C═N).	2.32 (s, 3H, CH_3_), 2.42 (s,3H, COCH_3_), 3.69 (s, 3H, CH_3_), 7.22–7.24 (d, 2H, Ar─H), 7.81–7.84 (d, 2H, phenyl ‐H), 5.5 (2H, NH_2, D2O exchanged_), 11.4 (1H, NH _D2O exchanged_).	
*4‐(1‐(2‐(4‐methyl‐5‐(phenyldiazenyl)thiazol‐2‐yl)hydrazineylidene)ethyl)aniline (5a)*.	3345, 3215(br.) (NH, NH_2_), 1589 (C═N).	2.43 (s,3H, CH_3_), 2.51 (s,3H, CH_3_)_,_ 5.79 (s,2H, NH_2_),6.61‐6.63 (d, 2H, phenyl ‐H), 7.34–7.39 (m, 5H, phenyl ‐H), 7.74–7.76 (d, 2H, phenyl‐H), 10.52 (s,1H, NH).	15.17(CH_3_), 16.50(CH_3_), 113.57, 116.01, 124.54(2C), 125.82(2C), 129.10 (2C), 129.60 (2C) 139.94(2C), 152.22, 165.18, 169.35(C─NH_2_), 177.14 (C═N).
*4‐(1‐(2‐(5‐((4‐chlorophenyl)diazenyl)‐4‐methylthiazol‐2‐yl)hydrazineylidene) ethyl)aniline (5b)*.	3339, 3239, 3202 (NH, NH_2_), 1676 (C═N).	2.19 (s, 3H, CH_3_), 2.51 (s,3H, CH_3_), 4.19 (s,2H, NH_2_) 6.56–8.02 (m,2H, phenyl‐H) 10.88 (s,1H, NH).	
*4‐(1‐(2‐(5‐((4‐methoxyphenyl)diazenyl)‐4‐methylthiazol‐2‐yl)hydrazineylidene) ethyl)aniline (5c)*.	3300, 3196 (br.) (NH, NH_2_), 1676(C═N). ^1^	2.19 (s, 3H, CH_3_), 2.48 (s, 3H, CH_3_) 3.72 (s, 3H, CH_3_), 4.18 (s, 2H, NH_2_), 6.53–6.57 (d, 2H, phenyl–H), 6.89–6.91 (d, 2H, phenyl–H), 7.27–7.30 (m, 2H, phenyl–H), 7.62–7.64 (m, 2H, phenyl–H), 10.87 (s, 1H, NH).	14.09 (CH_3_), 14.43 (CH_3_),, 55.75(CH_3_),, 114.96, 115.40, 115.66, 116.81, 127.44, 127.56, 128.60(2C), 135.02 (2C), 138.0, 144.64, 149.91, 154.77, 165.93, 192.89 (C═N),

### 2.2 Anticorrosive Analysis

#### 2.2.1 Potentio‐Dynamic Polarization (PDP) Estimation

**FIGURE 1:**
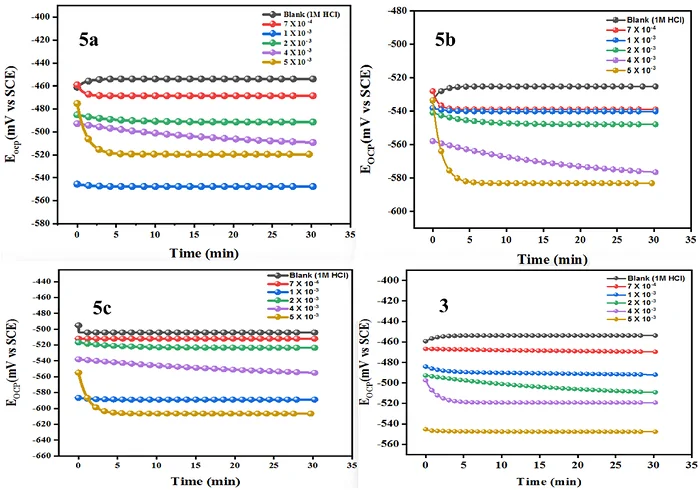
OCP Curves for the carbon steel (CS) electrode in 1 M HCl with different conc. of **3, 5a, 5b,** and **5c** compounds at room temperature.

Potentio‐dynamic polarization experiments were used to evaluate the carbon steel (CS) surface's resistance to corrosion in 1 M HCl. Both with and without produced **3, 5a, 5b**, and **5c** compounds from various solvents (blank). Prior to polarization, the (OCP) was recorded until a steady‐state value was established Figure 1 [27, 28]. With potential shifts ranging from ±85 mV, the OCP measurements revealed very little difference between the inhibited and uninhibited systems. Such minor potential variations indicate that these organic additives do not drastically disrupt the overall electrochemical equilibrium of the system. The strength of the corrosion potential (*E*_corr_) values listed in Table 2 is further supported by this stability. Overall, our findings demonstrate how produced chemicals can effectively reduce corrosion while preserving the equilibrium of electrochemical systems.

**TABLE 2:** Polarization parameters of carbon steel (CS) in 1 M HCl containing varied conc. of **3**, **5a**, **5b**, and **5c** along with the appropriate inhibition efficiency (%IE).

Inh.	Conc.(M)	−*E*_corr_ (mV)	*i*_corr_ (µA cm^−2^)	−β_c_ (mV.dec^−1^)	*Β*_a_ (mV.dec^−1^)	*R*_p_ (Ω. cm)	CR _(mpy)_	*θ*	IE (%)
	**Blank**	**541**	**0.7182**	**165.89**	**129.21**	**97.52**	**8.23**	**——–**	**——–**
**3**	**7 × 10^−4^**	**542**	**0.3125**	**164.24**	**125.14**	**115.87**	**6.29**	**0.6958**	**69.58**
	**1 × 10^−3^**	**494**	**0.2273**	**151.54**	**125.44**	**135.82**	**3.85**	**0.7471**	**74.71**
	**2 × 10^−3^**	**526**	**0.1701**	**139.48**	**119.65**	**178.44**	**2.11**	**0.7955**	**79.55**
	**4 × 10^−3^**	**512**	**0.0818**	**130.74**	**112.01**	**201.47**	**1.08**	**0.8012**	**80.12**
	**5 × 10^−3^**	**509**	**0.0317**	**129.44**	**122.55**	**215.01**	**0.82**	**0.8299**	**82.99**
**5a**	**7 × 10^−4^**	**555**	**0.2599**	**152.91**	**108.34**	**110.87**	**4.99**	**0.5815**	**58.15**
	**1 × 10^−3^**	**564**	**0.0992**	**134.45**	**117.94**	**119.56**	**2.15**	**0.6331**	**63.31**
	**2 × 10^−3^**	**547**	**0.0791**	**128.22**	**102.8**	**137.48**	**1.35**	**0.7583**	**75.83**
	**4 × 10^−3^**	**451**	**0.0588**	**124.69**	**119.52**	**185.59**	**0.88**	**0.8221**	**82.21**
	**5 × 10^−3^**	**458**	**0.0233**	**124.78**	**122.25**	**217.73**	**0.52**	**0.8889**	**88.89**
**5b**	**7 × 10^−4^**	**569**	**0.3298**	**129.87**	**99.39**	**120.52**	**2.89**	**0.5008**	**48.08**
	**1 × 10^−3^**	**555**	**0.1132**	**125.43**	**110.87**	**144.14**	**1.75**	**0.7123**	**71.23**
	**2 × 10^−3^**	**576**	**0.0811**	**123.29**	**116.45**	**159.74**	**0.96**	**0.8283**	**82.83**
	**4 × 10^−3^**	**583**	**0.0458**	**121.14**	**109.74**	**195.87**	**0.64**	**0.8945**	**89.45**
	**5 × 10^−3^**	**562**	**0.0183**	**125.36**	**120.59**	**248.03**	**0.42**	**0.9389**	**93.89**
**5c**	**7 × 10^−4^**	**592**	**0.3399**	**134.91**	**100.25**	**150.17**	**2.59**	**0.6128**	**61.28**
	**1 × 10^−3^**	**584**	**0.1273**	**122.87**	**97.74**	**165.22**	**1.35**	**0.7983**	**79.83**
	**2 × 10^−3^**	**566**	**0.0701**	**129.28**	**102.65**	**201.74**	**1.00**	**0.8583**	**85.83**
	**4 × 10^−3^**	**559**	**0.0414**	**121.64**	**112.55**	**228.24**	**0.78**	**0.8920**	**89.20**
	**5 × 10^−3^**	**568**	**0.0103**	**125.64**	**120.27**	**256.93**	**0.32**	**0.9499**	**94.99**

The polarization results revealed that the addition of the investigated compounds significantly reduced the corrosion current density (*I*_corr_), indicating effective suppression of the corrosion process. Although slight shifts in *E*_corr_ were observed upon inhibitor addition, the displacement remained below the commonly accepted threshold of ±85 mV relative to the uninhibited solution. Therefore, the investigated compounds cannot be classified as purely anodic or purely cathodic inhibitors and are more appropriately considered mixed‐type inhibitors. Furthermore, noticeable changes were observed in both anodic (β_a_) and cathodic (β_c_) Tafel slopes. The variation of β_a_ indicates that the inhibitors influence the anodic dissolution of iron, while the modification of βc demonstrates their effect on the cathodic hydrogen evolution reaction. The simultaneous alteration of both anodic and cathodic branches suggests that the inhibitor molecules adsorb on active sites associated with both electrochemical reactions.

This behavior can be attributed to the presence of multiple adsorption centers, including nitrogen, sulfur, and π‐electron‐rich aromatic systems, which facilitate strong adsorption onto the carbon steel surface. The resulting protective film acts as a barrier that restricts charge transfer and reduces the availability of active sites for both iron dissolution and hydrogen reduction reactions. Consequently, the investigated compounds exhibit mixed‐type inhibition behavior with no pronounced preference toward either anodic or cathodic control [29]. To produce electrochemical factors, the Tafel extrapolation process was used. This the approach produced significant values, including anodic current potential (*E*_corr_), density (*I*_corr_), anodic (β_a_) and cathodic (β_c_) Tafel slopes, and inhibition efficiency (*IE*). Equation (1) was used to calculate inhibition efficiency based on the *I*_corr_ values [30]. All results are presented in Table 2, providing clear information regarding the inhibitor's effectiveness at reducing corrosion processes.

(1) $$IE=\frac{1-I_{corr}^{0}}{I_{corr}}$$

The parameter $I_{\mathrm{corr}}^{0}$ show the corrosion current density in the leave of the prepared compounds, while *I*_corr_​corresponds to the value measured in its appearance.

**FIGURE 2:**
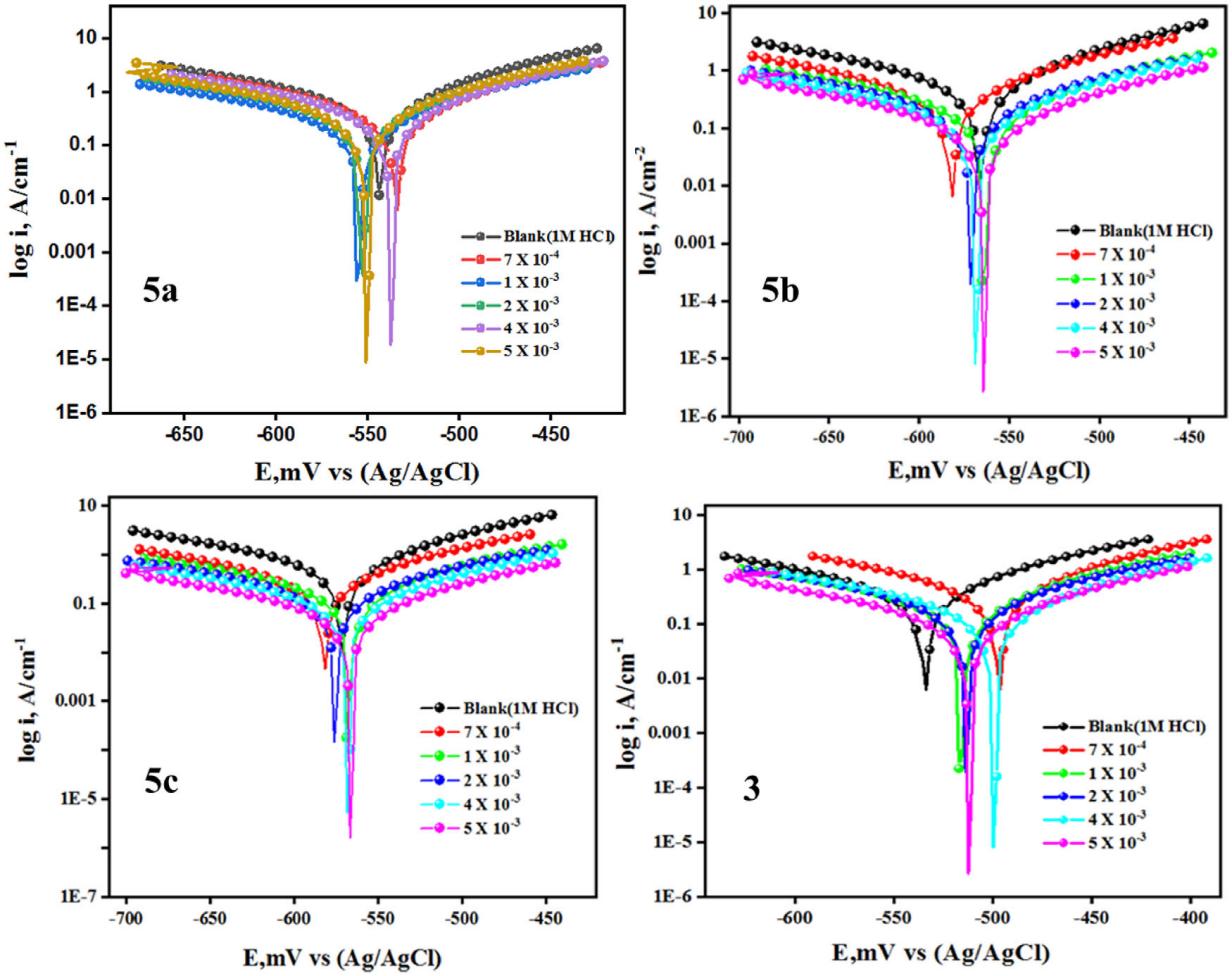
Polarization curves for the carbon steel (CS) surface in 1 M HCl with and without different conc. of **3**, **5a**, **5b**, and **5c** at 25C.

As the conc. of **3, 5a, 5b,** and **5c** compounds increased, both the (CR) and corrosion (*I*_corr_) results showed a significant increase in corrosion resistance, which varied depending on the solvent. At **5 × 10^−3 ^** M, the sequence of inhibitory efficiency is **5c** (95.10%) > **5b** (92.56%) > **5a** (89.01%) > **3** (83.41%) and supports the physical adsorption on the metal sample. This is because the thiazole derivatives donating ability to the metallic surface follows the order of **5c** > **5b** > **5a** > **3**. The usefulness of inhibitors was as follows: **5c** indicate the highest refinement, followed by **5a** and **5b** compounds and finally **3**. This maintains the inhibitor's ability to limit the breakdown of anodic metals. Additionally, it seems that cathodic hydrogen evolution contributes to protection by reducing the surface area that is directly exposed to the corrosive electrolyte [31]. As illustrated in Figure 2, the polarization curves support the mixed‐mode corrosion behavior. The polarization resistance (*R*_p_) was measurement using Equation (2), this provides more information on the inhibitor's protective ability.

(2) $$Rp=\frac{\beta a\;\times \;\beta c}{2.303\;i_{\mathrm{corr}}\left(\beta a+\beta c\right)}$$

#### 2.2.2 Electrochemical Impedance Spectroscopy

**FIGURE 3:**
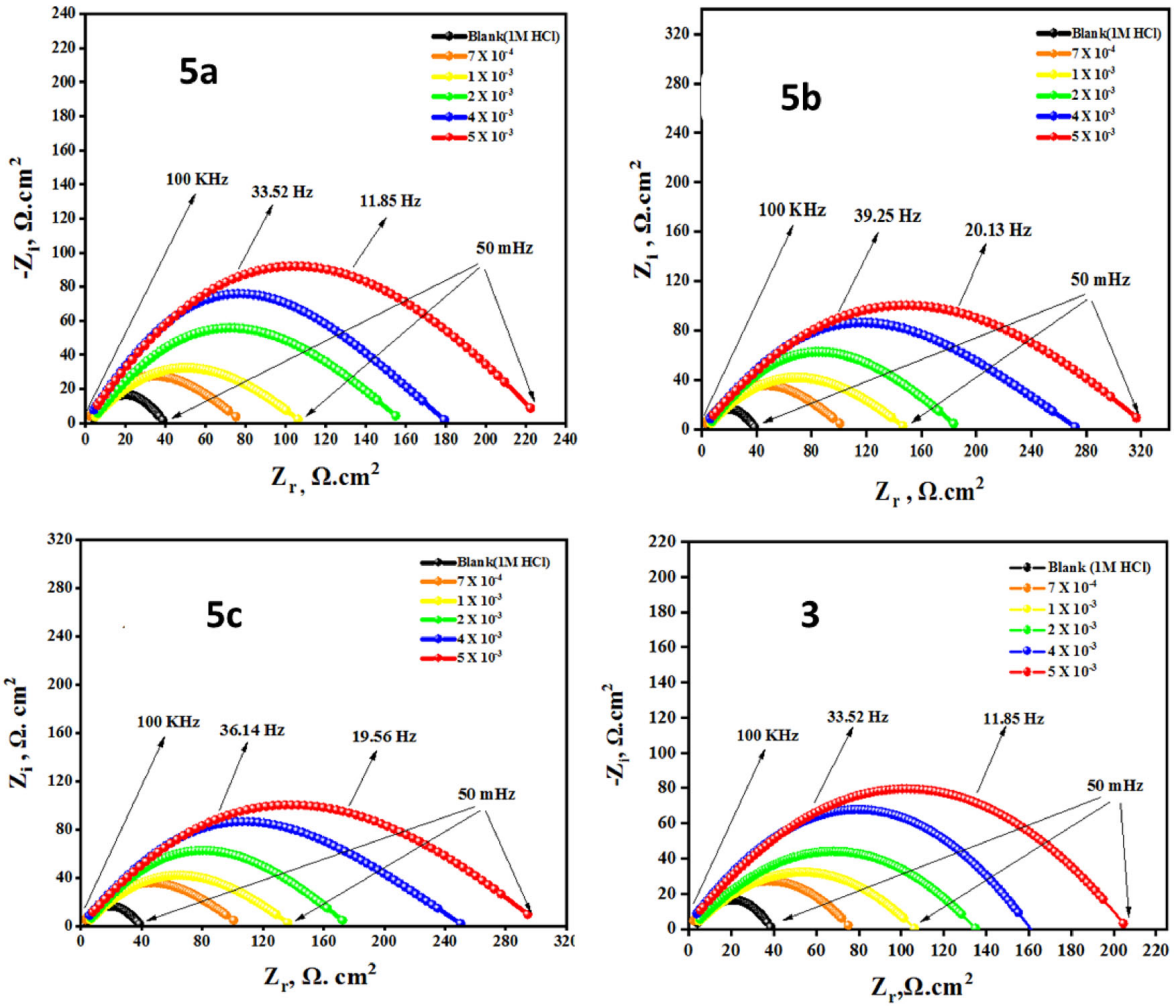
Nyquist plots for carbon steel (CS) surface added in 1 M HCl in the absence and presence of various concentrations of **3, 5a, 5b,** and **5c** at 25°C.

**FIGURE 4:**
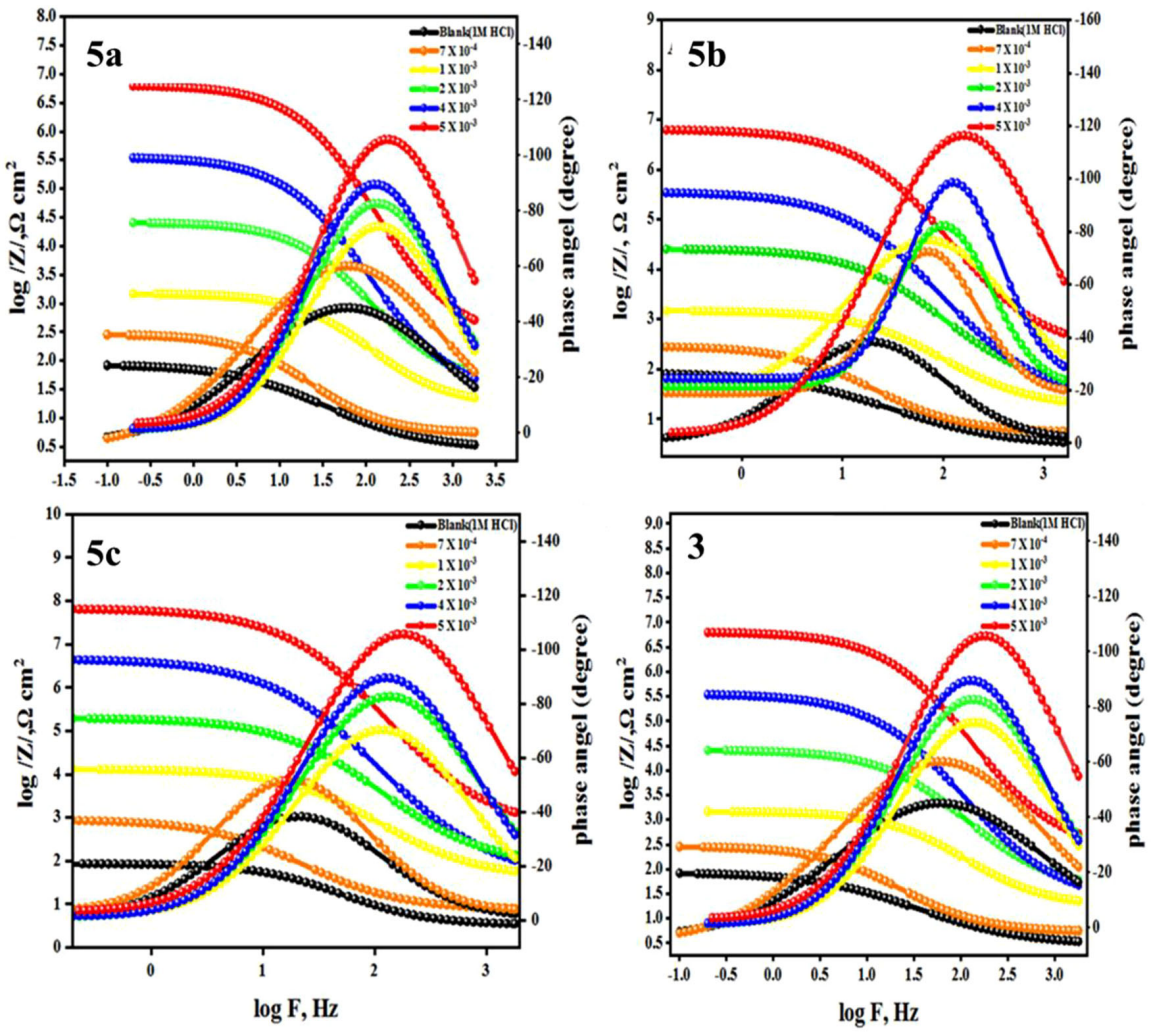
Bode and phase angle plots for the carbon steel (CS) surface in 1 M HCl solution, represent the effect of various conc. of **3, 5a, 5b,** and **5c** at 25°C.

In order to verify the electrochemical behavior that had been previously observed and to examine the capacitive characterization at the interface between carbon steel (CS) and the solution, electrochemical impedance spectroscopy (EIS) was employed. The Nyquist and Bode plots for CS samples submerged in 1 M HCl with and without altered concentrations of **3, 5a, 5b**, and **5c** are displayed in Figures 3 and 4. All of the cells' generated Nyquist plots have the same shape, suggesting that the addition of new substances to the corrosive acidic medium effectively lowers corrosion without changing the main electrochemical mechanism [32]. The semicircular arcs show at low frequencies indicate that charge transfer resistance dominates the corrosion process [33]. Moreover, Table 2 indicate higher exponent (*n*) values for compounds attended to with inhibitors compared to the blank, show that the compounds increase surface homogeneity via adsorption. Figure 4 indicate bode plots (log |Z| vs. log f) and phase angle curves that demonstrate this trend for CS in 1 M HCl, both in the absence and presence of inhibitors at different dosages [34]. The notable high in impedance magnitude |Z| at low frequencies established improved inhibition performance with high inhibitor concentration [35]. This refinement is assigned to the effective coverage of active surface sites by the adsorbed inhibitors. Therefore, the lowering in phase angle values propose that these compounds act by forming a preventive film on the CS surface [36]. The Equations (3)–(6) for calculating double‐layer capacitance (*C*_dl_) and inhibition efficiency (IE%) were as follows:

(3) $$\mathrm{IE}\%=\frac{\left(R_{\mathrm{ct}\left(inh\right)}^{0}-R_{\mathrm{ct}}\right)}{R_{\mathrm{ct}\left(inh\right)}}\;x\;100$$

The charge transfer resistances for both the untreated ($R_{ct}^{0}$) and inhibited (*R*_ct_) systems were intent according to the fitting of the Nyquist plots. Moreover, these resistances and the equivalent electrical circuit used for modeling inclusive a constant phase element (CPE), which accounts for the non‐ideal behavior of the electrochemical double layer [37].

The CPE is commonly used in state a pure capacitor to more rightly describe the frequency dispersion show in real electrochemical systems. The impedance associated with the CPE is linked to the double‐layer capacitance (*C*_dl_) [38] and was measurement using the following Equation (4).

(4) $$Z_{CPE}=\left[\frac{1}{Y_{0}{\left(j\omega \right)}^{n}}\right]$$

These parameters extend valuable insights into the surface environment and adsorption behavior of the new prepared inhibitors, with high *R*_ct_ values and decreased *C*_dl_ indicating enhanced surface preservation and active inhibitor performance.

The constant phase element (CPE) is described by its impedance, denoted as (*Z*_CPE_) which show its behavior within the electrochemical system. In this context, (*Y*_0_) represents the CPE constant (CPE), (*ω*) is the angular frequency, and the parameter (*j*) is linked in an imaginary number when n = 1, indicating a genuine capacitor, and at *n* = 0, indicating pure resistance impact. The CPE is inclusive in the system's double‐layer capacitance, as calculated by Equation (5).

(5) $$C_{dl}={\left(Y_{0}\;R_{\mathrm{ct}}^{1-n}\right)}^{1ln}$$

Table 3 summarizes the impedance parameters revealed by nonlinear least‐squares fitting. According to the research, there is a 90% improvement in corrosion resistance and a significant rise in charge transfer resistance (*R*_ct_) at greater concentrations of the newly created inhibitors. The creation of a dense and stable protective layer at the metal surface versus corrosive solution interface was demonstrated at a concentration of 5 × 10^−3^ M, which produced the highest inhibitory efficiency [39].

**TABLE 3:** Electrochemical impedance spectroscopy parameters and (IE%) inhibition efficiency for carbon steel in 1 M HCl solution of **3**, **5a**, **5b**, and **5c** conc. at 25°C.

Inh.	Conc.(M)	*R*_s_ (Ω cm^2^)	*R*_ct_ (Ω cm^2^)	*C*_dl_ (F cm^−2^)	*Y*_0_ (Ω^−1^ cm^−2^)	*χ* ^2^	*n*	*θ*	IE (%)
	Blank	2.56	36.25	88.32	242 × 10^−6^	0.00328	0.78	——–	——‐
3	7 × 10^−4^	3.02	116.24	81.47	212 × 10^−6^	0.00397	0.61	0.6089	60.89
	1 × 10^−3^	3.14	138.47	72.32	178 × 10^−6^	0.00454	0.59	0.6941	69.41
	2 × 10^−3^	3.45	185.91	64.78	112 × 10^−6^	0.00644	0.50	0.7474	74.74
	4 × 10^−3^	4.34	230.47	58.25	85 × 10^−6^	0.00568	0.68	0.8126	81.26
	5 × 10^−3^	5.67	298.55	51.85	64 × 10^−6^	0.00524	0.55	0.8341	83.41
5a	7 × 10^−4^	3.66	138.24	72.57	215 × 10^−6^	0.00412	0.59	0.6922	69.22
	1 × 10^−3^	3.14	168.14	69.55	180 × 10^−6^	0.00511	0.66	0.7532	75.32
	2 × 10^−3^	3.45	201.95	79.27	122 × 10^−6^	0.00652	0.45	0.8265	82.65
	4 × 10^−3^	4.94	295.14	54.55	92 × 10^−6^	0.00601	0.72	0.8514	85.14
	5 × 10^−3^	6.77	339.25	43.25	64 × 10^−6^	0.00594	0.69	0.8901	89.01
5b	7 × 10^−4^	3.45	146.36	78.36	210 × 10^−6^	0.00399	0.62	0.6347	63.47
	1 × 10^−3^	4.21	175.45	69.25	135 × 10^−6^	0.00400	0.54	0.7684	76.84
	2 × 10^−3^	3.94	210.78	72.48	98 × 10^−6^	0.00495	69.45	0.8369	83.69
	4 × 10^−3^	4.88	299.85	55.14	64 × 10^−6^	0.00562	0.48	0.8878	88.78
	5 × 10^−3^	7.56	356.47	49.27	59 × 10^−6^	0.00524	0.56	0.9256	92.56
5c	7 × 10^−4^	3.22	148.69	79.45	212 × 10^−6^	0.00450	0.70	0.6524	65.24
	1 × 10^−3^	3.97	186.47	75.47	178 × 10^−6^	0.00535	0.61	0.7821	78.21
	2 × 10^−3^	4.56	240.12	63.89	112 × 10^−6^	0.00625	0.54	0.8569	85.69
	4 × 10^−3^	4.35	301.89	59.24	85 × 10^−6^	0.00681	0.59	0.8947	89.47
	5 × 10^−3^	8.78	370.87	51.47	67 × 10^−6^	0.00624	0.42	0.9510	95.10

The use of a CPE instead of an ideal capacitor account for surface heterogeneity, roughness, and nonuniform current distribution at the electrode surface. The showed decrease in CPE values after inhibitor addition suggests a reduction in the local dielectric constant and/or an increase in the thickness of the electrical double layer due to the adsorption of inhibitor compounds. These findings support the formation of a compact protective layer on the carbon steel surface, which acts as a barrier against aggressive chloride ions and suppresses both anodic and cathodic reactions. The simultaneous increase in *R*_ct_ and decrease in CPE confirm the effectiveness of the investigated compounds as corrosion inhibitors [39].

Consequently, the electron transfer rate is reduced, as the development of the electrical double layer becomes increasingly hindered. This reduction in electron mobility contributes to the observed decrease in the corrosion rate (CR). The changes in double‐layer capacitance can be explained using the Helmholtz model, as reported in Equation (6) [40].

(6) $$\;C_{\mathrm{dl}}=\frac{\varepsilon_{0}\varepsilon_{s\;A}}{d}$$

where (*A*) appear for the cathode surface area, (*d*) is the thickness of the double layer, (*ε*_s_) denotes the dielectric constant of the medium, and (*ε*_0_) mention to the permittivity of free space (air). The phase change value (n) was found to range between 0.78 and 0.42, indicating effective protection of the metal surface against corrosion by limiting charge exchange between the surface of metal and the electrolyte ions, A smaller χ^2^ value indicates a closer match between the experimental spectra and the fitted equivalent‐circuit model, and therefore reflects greater confidence in the derived circuit parameters reported in Table 3, and confirms that the selected equivalent circuit is an appropriate physical model for the corrosion/inhibition process at the carbon steel against HCl media [41, 42].

#### 2.2.3 Adsorption Isotherm

The adsorption isotherm is frequently used to quantify inhibitor adsorption, and it provides essential information about the interactions between the inhibitor and the metal surface [43]. As the degree of metal surface covered (*θ*) by inhibitor compounds rose, the rate of corrosion actually reduced. Equation (7) can be used to determine *θ* since IE% is supposed to be a function of surface coverage [44]:

(7) $$\theta =10^{-2}XIE\%$$

The results showed that the Langmuir adsorption isotherm give the best fits. The Langmuir adsorption isotherm is reported in Equation (8) [43]:

(8) $$\frac{c}{\theta }=\frac{1}{K_{ads}}+c$$

where *K*_ad_ is the equilibrium constant of the adsorption process, *θ* is the surface coverage, and *C* is the inhibitor's molar concentration. Equation (9) was used to determine the free energy of adsorption (Δ*G*°_ads_) [45]:

(9) $$\Delta G_{\text{ads}}^{\circ }=-\text{RT ln}\left(55.5K_{\text{ads}}\right)$$

where *R* is the universal gas constant (8.314Jmol^−1^K^−1^), *T* is the absolute temperature (*K*), and the molar concentration of water is 55.5. M^−1^.The value of Δ*G*°_ads_ indicates the type of adsorption process. If Δ*G*°_ads_ was less than −20 kJmol^−1^, physisorption happened by electrostatic interactions; if Δ*G*°_ads_ was higher than −40 kJmol^−1^, chemisorption might happen via electron transfer from inhibitor molecules to the metal. The adsorption power, ∆*H*° _ads_, can be calculated using the van't Hoff equation, and the standard entropy of ∆*S*°_ads._ Equation (10) for adsorption can be calculated using the following thermodynamic equation.

(10) $$\Delta G_{\mathrm{ads}}^{\circ }=\Delta H_{\mathrm{ads}}^{o}-T\;\Delta S_{\mathrm{ads}}^{o}$$

The inhibitor's spontaneous nature on metallic surfaces is indicated by the negative Gibb's free adsorption energy values. In a more general sense, values exceeding − 40 kJ/mol promote chemisorption, which is defined by chemically interacting between metallic ions and inhibitor atoms, while values around or below − 20 kJ/mol are consistent with physisorption mediated by electrostatic interactions [46]. As shown in Table 4, the negative Δ*G*_ads_ confirms the stability and spontaneity of the adsorption process occurring at the metallic surface's edge. This finding is particularly in line with the widespread use of chemical adsorption. These results are consistent with the weight loss results, where calculate the −Δ*G*°_ads_ (− 20 kJ/mol‐< −Δ*G*°_ads_ > − 40 kJ/mol) for **3, 5a**, **5b,** and **5c** indicating the presence of physisorption processes and the **5c** shows better coverage. The thiazoles molecules **3, 5a**, and **5b** spontaneously adhered to the carbon steel surface as a result of −Δ*G*°ads Table 4 shows that inhibitor **5c** causes values of −Δ*S*°_ads_ to shift toward fewer negative levels. This shows that hydrogen ions are being emitted in order to produce adsorbed hydrogen atoms. The release of hydrogen ions will be presented down by inhibitor molecules adhering to the metal surface. As a result, the system's structure becomes less ordered instead than more organized, which lowers the activation entropy levels.

**TABLE 4:** Langmuir adsorption isotherm parameter on metallic surface with thiazoles under investigation in 1 M HCl.

Inhibitors	Temp (°C)	*K*_ad_ (M^−1^)	−Δ*G*°_ads _kJmol^−1^	−Δ*H*°_ads _kJmol^−1^	−Δ*S*°_ads _kJmol^−1^
3	25	975.779	−27018.2	−2.98935	−90.6094
	35		−27924.4	−2.98935	−93.6488
	45		−28830.6	−2.98935	−96.6882
5a	25	1600.18	−28244.3	−3.20417	−94.7211
	35		−29191.6	−3.20417	−97.8984
	45		−30138.9	−3.20417	−101.076
5b	25	3073.322	−29862.1	−3.48761	−100.146
	35		−30863.7	−3.48761	−103.506
	45		−31865.2	−3.48761	−106.865
5c	25	3792.016	−30383	−3.57887	−101.893
	35		−31402	−3.57887	−105.311
	45		−32421.1	−3.57887	−108.729

### 2.3 Surface Morphology Examination

#### 2.3.1 Scanning Electron Microscopy (SEM)

**FIGURE 5:**
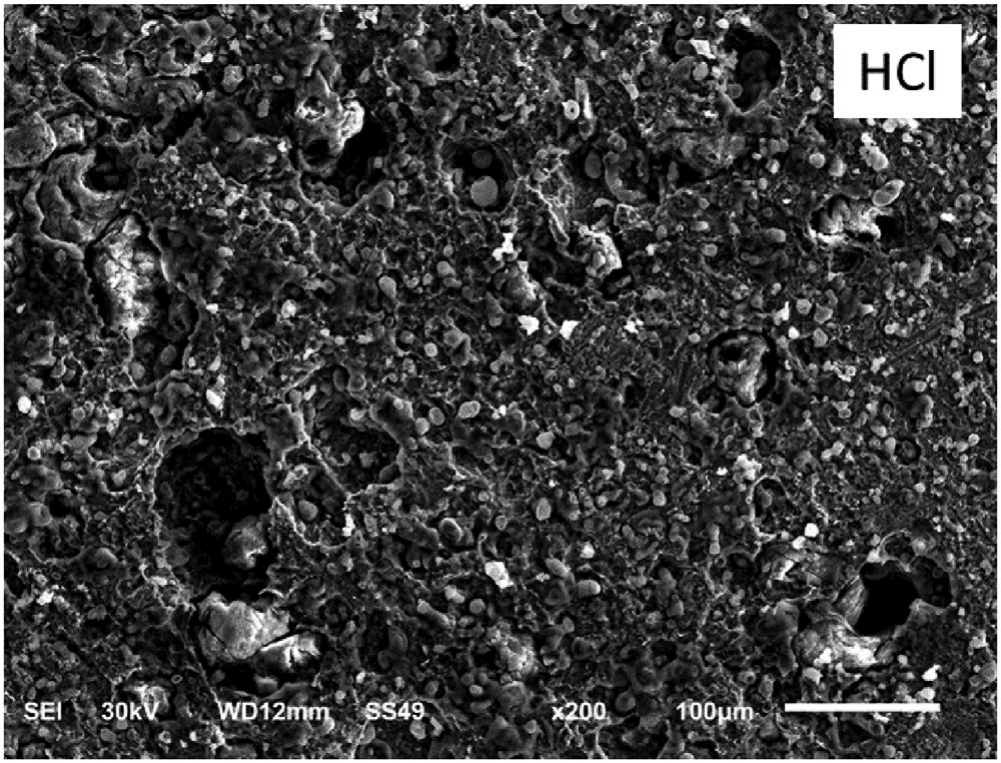
SEM micrographs of the carbon steel surface immersed in 1 M HCl.

**FIGURE 6:**
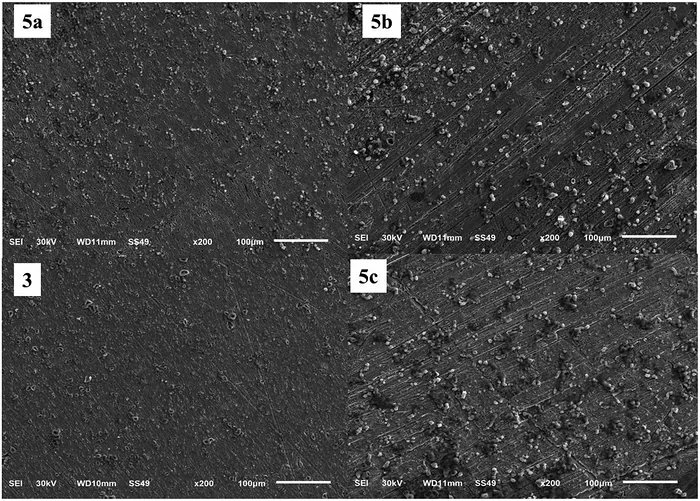
SEM micrographs of the carbon steel surface immersed in 1 M HCl in the absence and presence of different prepared inhibitors at various conditions.

Scanning electron microscopy (SEM) has been used to examine the morphology of the CS surface. In Figures 5 and 6, SEM picture of the surface of polished carbon steel. The micrograph displays a distinctive inclusion that was most likely an oxide inclusion. The SEM observations strongly corroborate the electrochemical findings, confirming that the prepared inhibitors protect the carbon steel surface through adsorption and formation of a dense protective layer that blocks active corrosion sites. All samples (HCl, **3, 5a, 5b,** and **5c**) were immersed in the solution for a specific period. After immersion, the HCl sample showed a rough and severely damaged surface, whereas the samples treated with **3, 5a, 5b,** and **5c** exhibited smoother surfaces, confirming development of a protective layer on the steel surface.

The SEM micrograph of the blank sample (HCl 1 M) exhibits severe surface degradation characterized by extensive localized corrosion, deep pits, and nonuniform corrosion products. In contrast, the steel surfaces treated with **5a** and **5b** inhibitors treated surfaces showed reduced pit density and more homogeneous surface textures compared to the blank. The **5c** specimen displayed further improvement, with fewer corrosion features and enhanced surface uniformity. Remarkably, the **3** sample demonstrated the smoothest surface with minimal damage, indicating the formation of a dense, compact, and uniform protective film.

#### 2.3.2 Adsorption Mechanism and Surface Complex Formation

The significant corrosion prevention efficacy of the thiazole ligands (**3, 5a, 5b,** and **5c**) is due to its ability to form a fixed surface complex with carbon steel through adsorption and coordination interactivity. The chemical structure of thiazole ligands has a number of electrons‐donating sites, including the azo nitrogen (─N═N─), acetyl (COMe), methoxy (OMe), and amine (NH_2_). These facilities allow strong donor–acceptor connection with vacant iron‐d orbitals. A tight and sticky protective coating is created when thiazoles ligand molecules adsorb onto the carbon steel surface and create a coordinated Fe–thiazoles ligands surface complex while submerged in hydrochloric acid solution. By effectively isolating the carbon steel surface from harmful Cl^−^ ions and dissolved oxygen, this surface complex inhibits both anodic dissolution and cathodic reduction processes

#### 2.3.3 Quantum Chemistry Calculations

##### 2.3.3.1 Molecular Modeling

**FIGURE 7:**
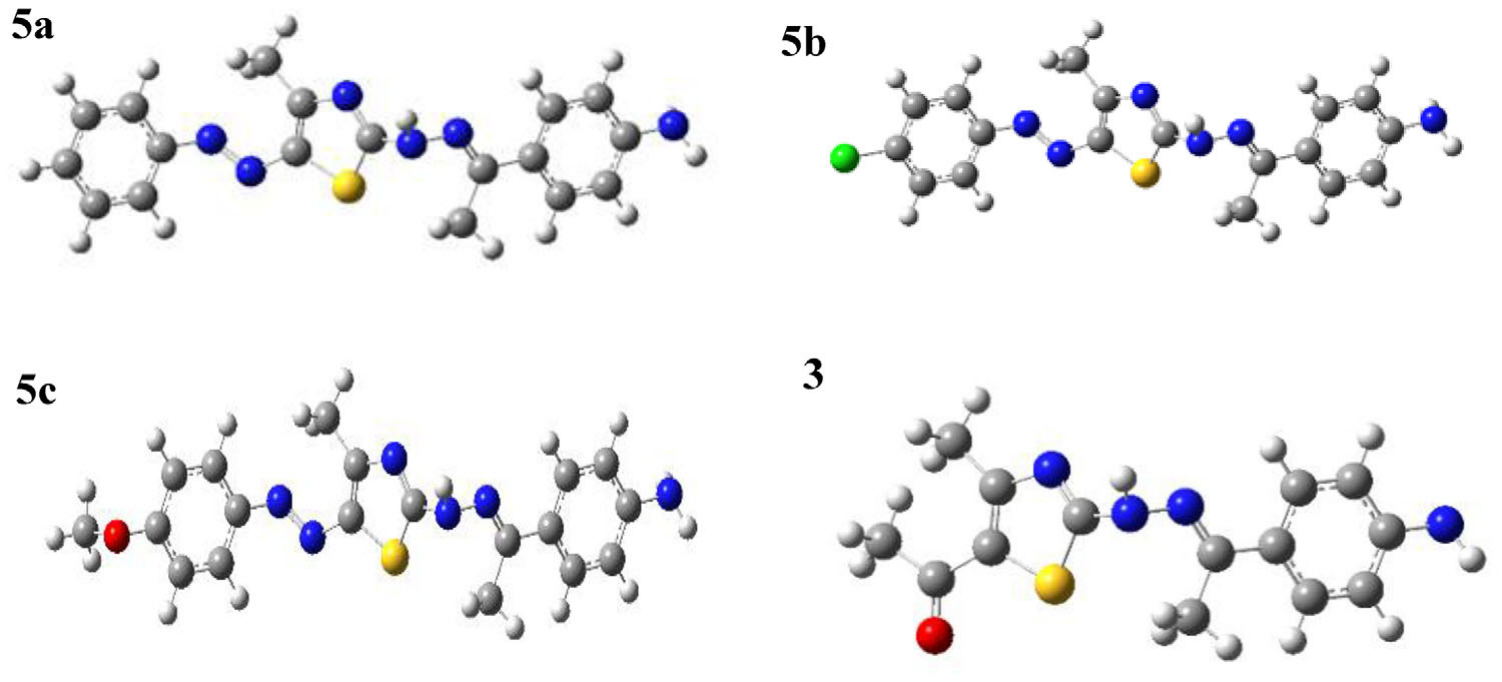
Optimized atomic orbital, numbering scheme, and geometry of the compound's border molecular orbital **5a, 5b, 5c, and 3**.

**FIGURE 8:**
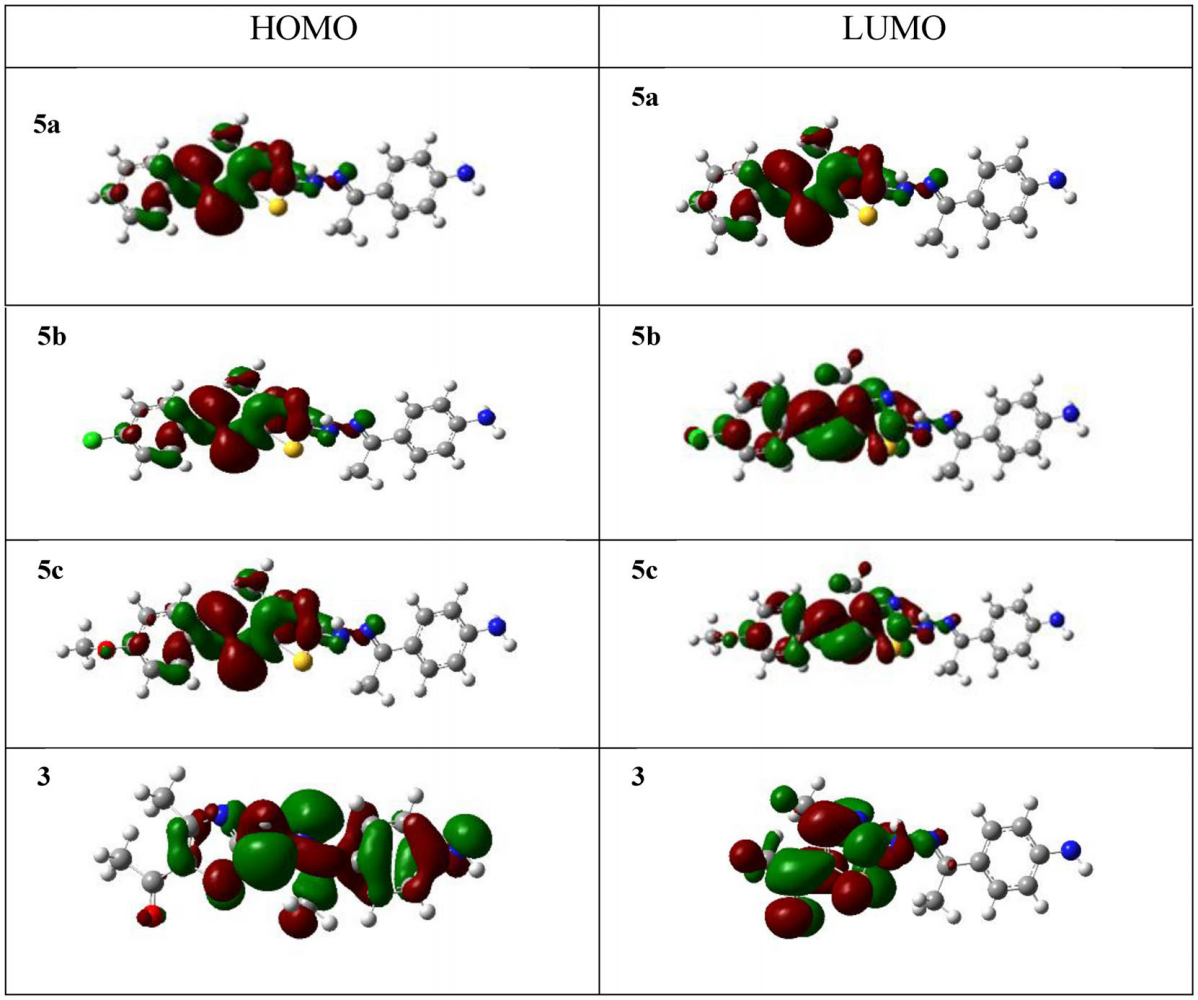
HOMO and LUMO orbital geometry for **3, 5a, 5b, and 5c**.

Density functional theory (DFT) computations were performed on the (**3, 5a, 5b,** and**5c**) sensitizers using the B3LYP/6‐31G (d,p) [47]. The optimal structure for compounds (**3, 5a, 5b,** and **5c**) is depicted in Figure 7 and clarify the connection between the geometric structure of the (**3, 5a, 5b,** and**5c**) sensitizer dyes. Frontier molecular orbital (FMO) analysis was conducted to assess the highest occupied molecular orbital (HOMO) and the lowest unoccupied molecular orbital (LUMO), which play a crucial role in determining chemical reactivity. The LUMO indicates its capacity to accept electrons, while the HOMO reflects the ability of a molecule to donate electrons. The electrical distribution of their LUMO and HOMO levels in further detail is shown in Figure 8.The energy difference between these orbitals (Δ*E* = *E*_LUMO_—*E*_HOMO_) is a key parameter for understanding the stability and reactivity of the compounds in Table 5 which exhibit the computed values for compounds **5a 5b, 5c,** and **3** *E*_HOMO_, *E*_LUMO_, and energy gap (Δ*E*). The highest occupied molecular orbitals are shown by the HOMO energy values (*E*_HOMO_), which vary from −5.47 to −5.9 eV. The energy levels of the lowest empty orbitals are represented by the (*E*_LUMO_) energy values (*E*_LUMO_), which range from −2.2 to −3.79 eV. More negative *E*_LUMO_ values indicate a higher capacity to take electrons.

**TABLE 5:** Geometrical optimization and spatial dispensation orbitals of derivative **3, 5a, 5b,** and **5c**.

Compounds	*E*_LUMO_ (ev)	*E*_HOMO_ (ev)	Δ*E* = *E*_LUMO_–*E*_HOMO_
3	−2.2	−5.9	1.88
5a	−3.6	−5.47	1.87
5b	−3.79	−5.64	1.85
5c	−3.62	−5.5	1.99

The HOMO–LUMO energy map showed in Figures 7 and 8. Greater molecule stability is indicated by greater HOMO–LUMO energy gaps (Δ*E*), which vary from 1.85 to 1.99 eV. Consequently, compared to other derivatives, derivatives **5c** (Δ*E* = 1.99) have greater molecular stability and reactivity. The donor portions (thiazole ring) and the hydrazono (NH─N═) attached to the benzene ring were where the HOMO electron density was primarily found for the **5c** sensitizer, whereas the 4‐methoxybenzene ring was where the LUMO electron density was primarily found. However, in **5a** and **5b**, the thiazole ring and the connecting hydrazono bridge (─NH─N═) and also the 4‐substituted (**5b**, ex: Cl) were the primary locations of the LUMO electron density. On the other side of the molecule, namely on the thiazole ring, methyl group (CH_3_), and azo linker (─N═N─) attached to the benzene ring, the electron density of HOMO of **3** was concentrated.

The FMO theory claims that the *E*_HOMO_ and *E*_LUMO_ values characterize the inhibitor molecules' acceptance or donation proficiency at the metal–inhibitor contact [48]. The molecules with a lower energy gap (Δ*E*) are therefore assigned to have a higher tendency toward adsorption since it shows that only a small amount of energy is required for the electron transition from *E*_HOMO_ to *E*_LUMO_ [49]. This is because the growth in *E*_HOMO_ values and the decrease in *E*_LUMO_ values increase the inhibition capability. As stated by our tabulated data, the Δ*E* values for (**5a, 5b**, and **5c**) are 1.87, 1.85, and 1.88 eV, respectively. These results are roughly the same but significantly different from the 3.70 allocated for structure **3**. The inclusion of the azo linkage in addition to the benzene ring increases the inhibitory efficacy of our synthesized structures, as seen by the Δ*E* data (Table 6), which prefer the adsorption of (**5a, 5b**, and **5c**) on the carbon‐steel surface over structure **3**. As the donation activity rises with decreasing electronegativity values, the electronegativity (χ) values also indicate the inhibition efficiency Equation (14) [50]. The electronegativity of structure **3** is 4.05 which is significantly lower than for **5a, 5b, 5c** which is 4.54, 4.72, and 4.56 respectively.

(11) $$\mu =-\chi =-\frac{\mathrm{Ip}+E_{a}}{2}$$

(12) $$\sigma =-\frac{1}{\eta }$$

(13) $$\eta =-\frac{\mathrm{Ip}+E_{a}}{2}$$

(14) $$\chi =\frac{\mathrm{Ip}+E_{a}}{2}$$

**TABLE 6:** Showing DFT‐related inhibitor parameters **3**, **5a, 5b,** and **5c**.

Quantum parameters	3	5a	5b	5c
Δ*E*, eV	3.70	1.87	1.85	1.88
Ionization potential (*I*)	5.90	5.47	5.64	5.50
Electron affinity (*A*)	2.20	3.60	3.79	3.62
Electronegativity (*χ*) eV	4.05	4.54	4.72	4.56
global hardness (*η*) eV	1.85	0.94	0.93	0.94
Softness (*σ*) eV^−1^	0.54	1.07	1.08	1.06
*ω*	4.43	11.00	12.02	11.06
Δ*Ν*	0.80	1.32	1.24	1.30
Δ*E*_back‐donation_	−0.46	−0.23	−0.23	−0.24

Δ*N* is related to these parameters via the Pearson method in Equation (15).

(15) $$\Delta N=\frac{\chi_{Fe}-\chi_{inh}}{2\left(\eta_{Fe}-\eta_{inh}\right)}$$

The global hardness (*η*) and softness (*δ*) confirm the stability and that inhibitors undergo chemical change and chemical ionization potential (*μ*) were calculated using the following formulas in terms of *I*_P_ and Ea (Equation (11), (12), (13)), (Table 6) [51]. It is known that as softness increases and hardness decreases, this confirms the high reactivity, which is attributed to the fluent donation of electrons from the inhibitors to the carbon steel surface [52]. Structure **3**'s softness value is 0.54, whilst the values for (**5a, 5b**, and **5c**) are 1.07, 1.08, and 1.06. In contrast, structure **3's** hardness value is 1.85, but the values for (**5a, 5b**, and **5c**) are 0.94, 0.93, and 0.94. Structure **3** is the hardest on electronic transitions, according to the softness and hardness calculations. According to the Table 6, structure **3** has the lowest Δ*N* (0.80), which indicates that it has the lowest electron contributing competency. Δ*N* is a measure of a molecule's capacity to give electrons [53]. The transferred electrons will be back‐donated when Δ E _back donation_ is less than zero, indicating the highest energetic inhibitory capacitance associated with a significant electron contribution [54]. Our theoretically computed values indicate that structures **5a, 5b,** and **5c** are better inhibitors than structure **3,** which may be explained by the Azo group's presence, which creates additional adsorption active centers. Additionally, when combined, they provide a large cloud of delocalized π‐electrons that may easily coordinate with the metal surface. The theoretically calculated parameters give indication that the inhibition efficiency is very close to each other which is accurate but it ranks them as follows: **5b > 5a > 5c** while our experimental results arrange them as follows:**5c >5 b >5a**. This issue is readily resolved by realizing that the presence of the bulkier but planar –OMe group, with the ring allowing to cover a larger surface area, is responsible for the lead inhibition effectiveness of inhibitor **5c**. This group offers an extra coordination site through the lone pair on the methoxy oxygen atom in addition to its bulkiness.

##### 2.3.3.2 Molecular Electrostatic Potential (MEP)

**FIGURE 9:**
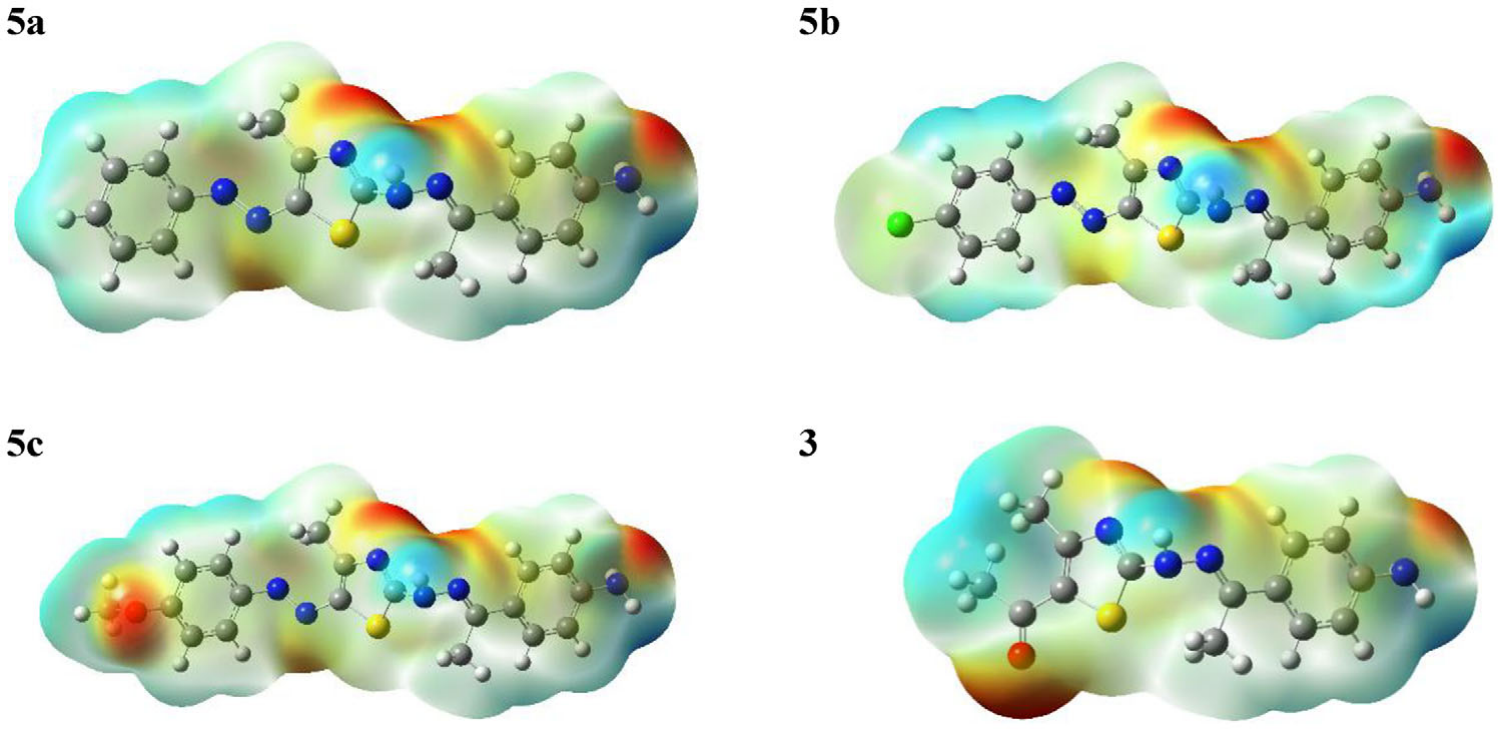
Molecular electronic potential diagram (MEP) of sensitizer  structure **3, 5a, 5b, and 5c**.

The HOMO and LUMO energy levels of compounds in (**3, 5a, 5b, and 5c**.) thiazole are examples of organic structure internal charge transfer (ICT) features that may be computed using molecular electrostatic potentials (MEPs), which are retrieved from a cube file produced using Gaussian calculations. The findings of examining the HOMO–LUMO levels and MEPS of all sensitizers **3, 5a, 5b,** and **5c** to ascertain the donor‐acceptor group effect are shown in Figure 9. The blue areas of the MEP, which are electron‐deficient sensitizer regions (**3, 5a, 5b, and 5c**), show nucleophilic activity, while the red sections, which are electron‐rich regions, show electrophilic activity. The electrostatic potential grew in the order shown below: red, yellow, orange, green, and blue. For the **3** sensitizer, the negative (red) charge is primarily localized acetyl (COMe) group in thiazole ring and on the (NH_2_) amino group in benzene ring. In **5a**, the negative (red) low potential is mainly showed around methyl group in thiazole ring and (NH_2_) amino group in in benzene ring. Additionally, in **5b** and **5c**, the negative (red) low potential is mainly presented around chloro (Cl), methoxy (OMe) group in para position of the benzene ring and (NH_2_) amino group in benzene ring, respectively. The positive region (blue) of the MEP map is indicated across the azo group, indicating that it is favorable site for nucleophilic attack.

##### 2.3.3.3 Monte Carlo Stimulation

Monte Carlo stimulation was used to determine the adsorption strength and stable configuration arrangement of the investigated inhibitors on the carbon steel crystal. To determine the best adsorption locations and track how inhibitors interact with the surface area of the Fe (11 0) crystal, the adsorption locator module was stimulated [55]. The nearly parallel adsorption designs are shown in the Table 7, This increases the surface covered and demonstrates and inhibitory efficiency [56]. The calculated adsorption energies, alongside the deformation and binding energies, are summarized in Table 8. Crucially, adsorption energy values were considerably negative for every inhibitor compound that was assessed. According to classical thermodynamics, the adsorption of these inhibitors onto the iron crystal is a spontaneous and energetically beneficial process as the energy value is negative [57]. All synthesized inhibitors have a thermodynamic attraction to move toward and coordinate with the carbon steel surface, as demonstrated by the simulation's results but ranked as follows (**5c > 5b > 3 > 5a**) specially attributed to dEad/dNi (kcal/mol−^1^) values which is −207.896 > −177.189 > −172.818 > −170.659, respectively. The experimentally obtained inhibitory efficiencies from electrochemical measurements did not strictly correlate with the ranking obtained from the computed adsorption energies in a linear fashion as it is ranked as follows(**5c >** **5b > 5a > 3**), this mismatch between the theoretical energy ranking and practical performance is a well‐documented phenomenon that arises from difference between an idealized atomistic simulation (that makes dynamic environmental influences easier to understand) and a complicated laboratory environment.(influenced with Competitive adsorption with solvent molecules, changes in solution pH, and the existence of native oxide coatings on carbon steel).

**TABLE 7:** Monte Carlo (MC) stimulation (displaying the inhibitors' adsorption on the Fe (1 1 0) crystal).

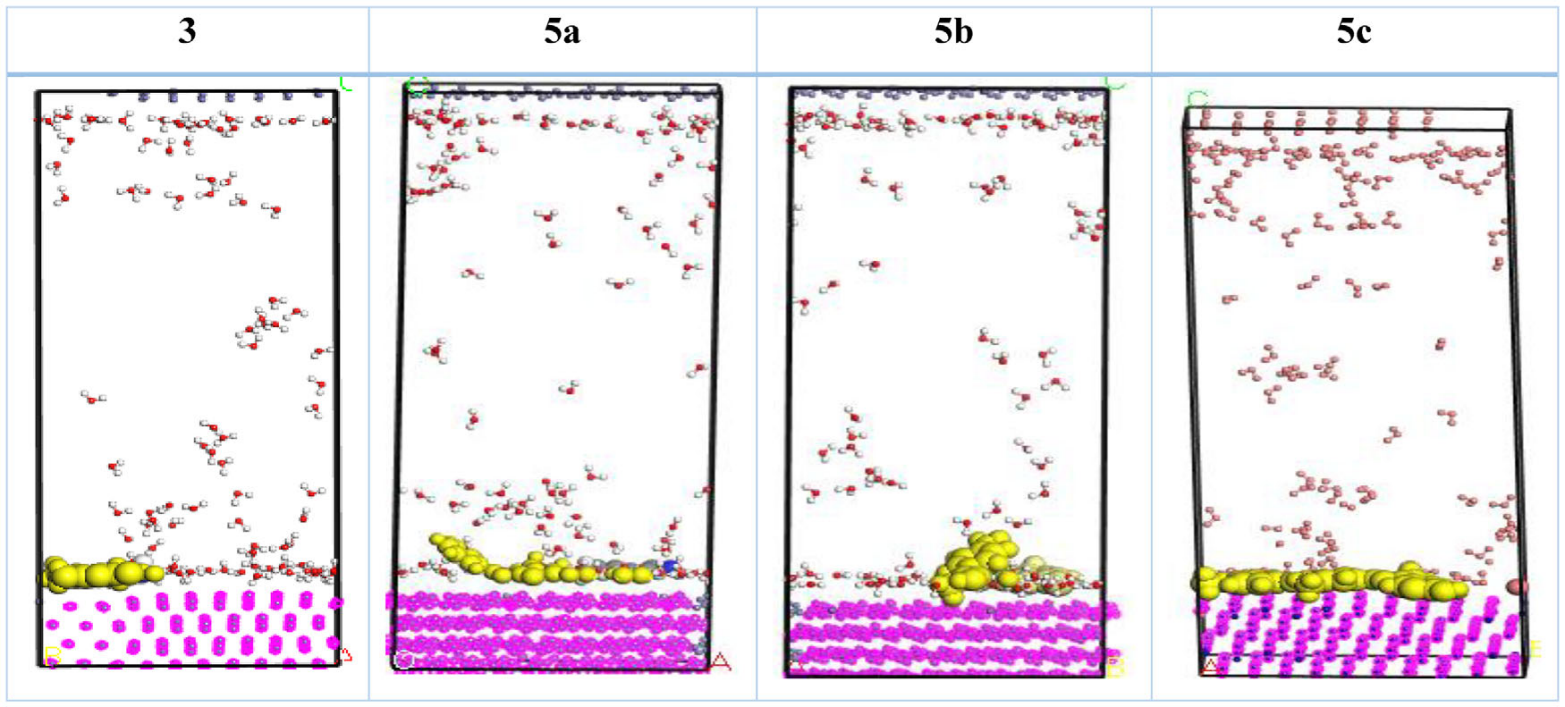

**TABLE 8:** Related parameters acquired via Monte Carlo stimulation.

Inh.	Total energy (kcal/mol)	Adsorption energy (kcal/mol)	Rigid adsorption energy (kcal/mol)	Deformation energy kcal/mol^−1^	dEad/dNi (kcal/mol^−1^)	Water: dEad/dNi (kcal/mol^−1^)
3	−1.221	−1.163	−1.216	53.577	−172.818	−0.303
5a	−1.189	−1.139	−1.194	54.551	−170.659	−0.221
5b	−1.258	−1.207	−1.254	46.476	−177.189	−0.440
5c	−1.183	−1.137	−1.184	46.561	−207.896	−0.278

### 2.4 Thiazole Derivative Inhibition Mechanism

**FIGURE 10:**
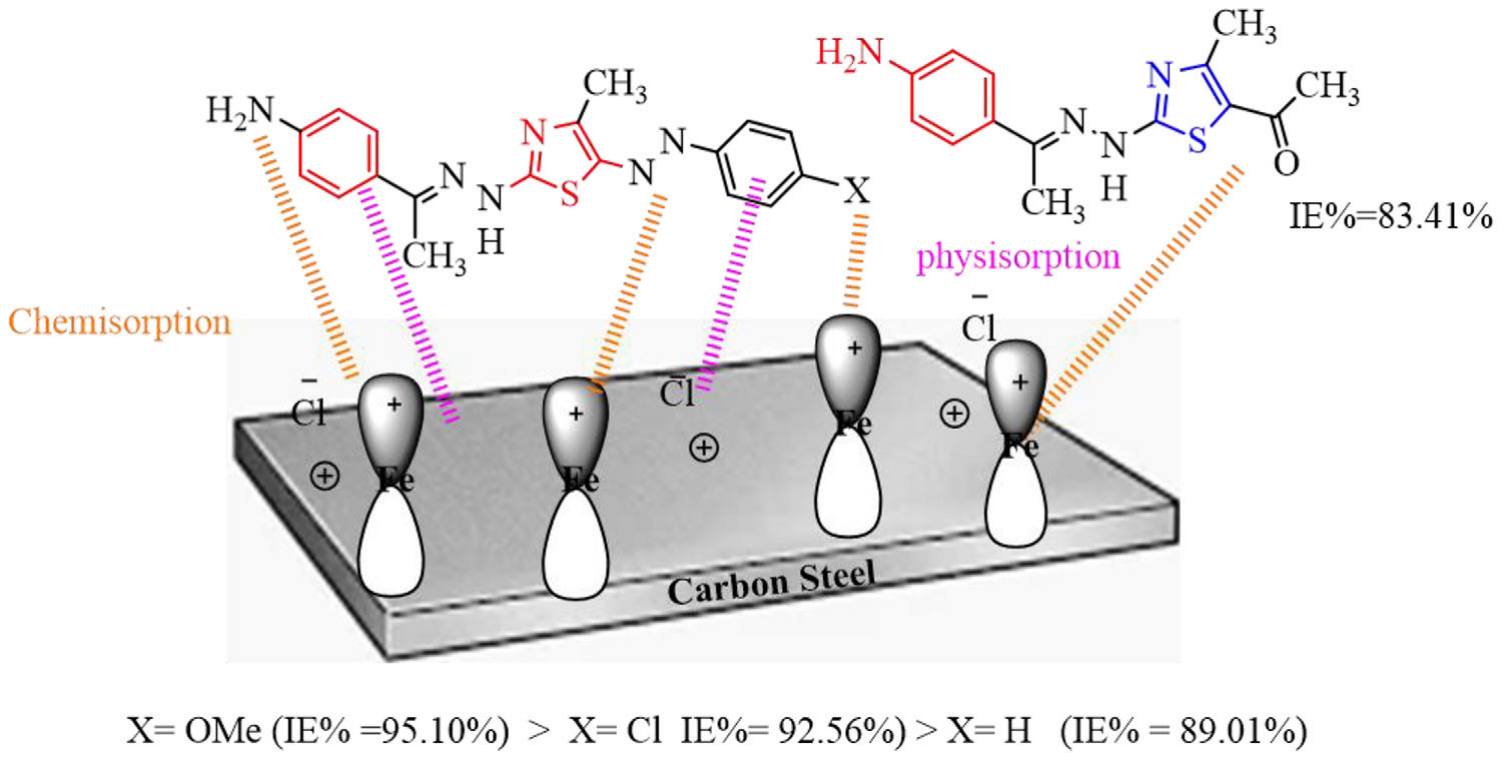
Adsorption mechanism of thiazoles on carbon steel surface in 1 M H Cl.

The inhibitor molecules' adsorption onto the carbon steel surface is influenced by a variety of parameters, including their atomic charges, chemical structure, and behavior in an acidic environment. The adsorption isotherm investigations also supported the Temkin adsorption [58] model's hypotheses on how the molecules **3, 5a, 5b,** and **5c** adsorb to the metallic substrate. Chemical adsorption onto the metallic substrate is facilitated by electron pairs of heteroatoms in the molecules **3, 5a, 5b,** and **5c** Figure 10 illustrates the proposed method for shielding corrosion‐resistant metallic substrates in an acidic medium. Because of their propensity to be adsorbed on the surface of carbon steel alloy by mutual chemisorption and physisorption, the thiazole derivatives showed good corrosion inhibition properties, as shown in Figure 10. The formation of coordinating bonds between the surface of the carbon steel alloy with empty Fe atom d‐orbitals and the lone pairs in thiazoles derivatives is known as chemisorption. As a result, the thiazoles molecules can create a strong, sticky adsorbed coating on the CS alloy's surface by a combination of chemical and physical interactions, shielding the surface from a harsh environment and preventing further dissolution.

## 3 Experimental Section

### 3.1 General Remarks

Melting points were determined on a Gallenkamp electric apparatus. IR spectra (KBr discs) were measured Thermo Scientific Nicolet iS10 FTIR spectrometer Also, Bruker's spectrometer 400 MHz, and JEOL spectrometer 500 and 400 MHz, (^1^H NMR), 125 and 100 MHz (^13^C NMR) were used to estimate NMR spectra in DMSO‐*d_6_* ^,^ as a solvent and an internal standard. A direct probe controller inlet connected to a single quadrupole mass analyzer in a Thermo Scientific GC–MS model ISQ LT was used to obtain EI–Mass spectra. Thermo X‐Calibur software was used to analyze the data at the Regional Center for Mycology and Biotechnology (RCMB) at Al‐Azhar University in Nasr City, Cairo. At Cairo University, elemental analyzes were performed using a PerkinElmer 2400 analyzer.

#### 3.1.1 Synthesis of 1‐(2‐(2‐(1‐(4‐Aminophenyl)Ethylidene)Hydrazineyl)‐4‐methylthiazol‐5‐yl)Ethan‐1‐one (**3**)

The mixture of the appropriated 3‐chloropentane‐2,4‐dione **2** (0.01 mol) with 2‐(1‐(4‐aminophenyl)ethylidene)hydrazine‐1‐carbothioamide **(1)** (2 gm, 0.01 mol) in 20 mL of EtOH /TEA (3 drops) was refluxed for 2–3 h and reaction ending was monitored by TLC. The resultant solid **3** was dried, and recrystallized from ethanol: DMF (1:2).

Green powder; yield 43%; mp 232°C; IR (KBr)*ν*/cm^−1^: 3182, 3054 (NH, NH_2_), 1732(C═O), 1619(C═N). ^1^H NMR (400 MHz, DMSO‐*d*_6_): *δ* (ppm): 2.32 (s,3H, CH_3_), 2.42 (s,3H, COCH_3_), 3.69 (s,3H, CH_3_), 7.22–7.24 (d, 2H, Ar─H), 7.81–7.84 (d, 2H, phenyl ‐H), 5.5 (2H, NH_2, D2O exchanged_), 11.4 (1H, NH _D2O exchanged_). Analy. Calcud. for Formula: C_18_H_18_N_6_S (350.44) Elemental Analysis: C, 61.69; H, 5.18; N, 23.98; Found: C, 61.67; H, 5.19; N, 23.96%.

### 3.2 General Procedure for the Synthesis of 4‐(1‐(2‐(4‐Methyl‐5‐(Aryl Diazenyl)Thiazol‐2‐yl)Hydrazineylidene)Ethyl)Aniline (**5a–d**)

The mixture of the appropriated 1‐chloro‐1‐(aryldiazenyl)propan‐2‐one **4a–c** (0.01 mol) with 2‐(1‐(4‐aminophenyl)ethylidene)hydrazine‐1‐carbothioamide **(1)** (2 gm, 0.01 mol) in EtOH /TEA (3 drops) was refluxed for 2–3 h and reaction finishing was monitored by TLC. The resultant solid **5a–c** was dried, and recrystallized from ethanol: DMF (1:2).

#### 3.2.1 4‐(1‐(2‐(4‐Methyl‐5‐(Phenyldiazenyl)Thiazol‐2‐yl)Hydrazineylidene)Ethyl)Aniline (**5a**)

Reddish orange powder; yield 43%; mp 130°C; IR (KBr)*ν*/cm^−1^: 3345, 3215(br.) (NH, NH_2_), 1589(C═N). ^1^H NMR (400 MHz, DMSO‐*d*_6_): *δ* (ppm): 2.43 (s,3H, CH_3_), 2.51 (s,3H, CH_3_)_,_ 5.79 (s,2H, NH_2_), 6.61‐6.63 (d, 2H, phenyl ‐H), 7.34‐7.39 (m, 5H, phenyl ‐H), 7.74–7.76 (d, 2H, phenyl‐H), 10.52 (s,1H, NH).^13^C NMR (δC/ppm):,15.17, 16.50, 113.57, 116.01, 124.54(2C), 125.82(2C), 129.10 (2C), 129.60 (2C) 139.94(2C), 152.22, 165.18, 169.35(C─NH_2_), 177.14 (C═N). Analy. Calcud. for Formula: C_18_H_18_N_6_S (350.44) Elemental Analysis: C, 61.69; H, 5.18; N, 23.98; Found: C, 61.67; H, 5.19; N, 23.96%.

#### 3.2.2 4‐(1‐(2‐(5‐((4‐Chlorophenyl)Diazenyl)‐4‐methylthiazol‐2‐yl)Hydrazineylidene) Ethyl)Aniline (**5b**)

Yellow powder; yield 43%; mp 226°C; IR (KBr)*ν*/cm^−1^: 3339, 3239, 3202 (NH, NH_2_), 1676 (C═N). ^1^H NMR (400 MHz, DMSO‐*d*_6_): *δ* (ppm): 2.19 (s,3H, CH_3_), 2.51 (s,3H, CH_3_), 4.19 (s,2H, NH_2_) 6.56–8.02 (m,2H, phenyl‐H) 10.88 (s,1H, NH). Analy. Calcud. for Formula: C_18_H_17_ClN_6_S (384.89) Elemental Analysis: C, 56.17; H, 4.45; N, 21.84; Found: C, 56.14; H, 4.43; N, 21.86%.

#### 3.2.3 4‐(1‐(2‐(5‐((4‐Methoxyphenyl)Diazenyl)‐4‐methylthiazol‐2‐yl)Hydrazineylidene) Ethyl)Aniline (**5c**)

Yellow powder; yield 43%; mp 232°C; IR (KBr)*ν*/cm^−1^: 3300, 3196 (br.) (NH, NH_2_), 1676(C═N). ^1^H NMR (400 MHz, DMSO‐*d*_6_): *δ* (ppm): 2.19 (s, 3H, CH_3_), 2.48 (s, 3H, CH_3_) 3.72 (s, 3H, CH_3_), 4.18 (s, 2H, NH_2_), 6.53–6.57 (d, 2H, phenyl–H), 6.89–6.91 (d, 2H, phenyl–H), 7.27–7.30 (m, 2H, phenyl–H),7.62–7.64 (m, 2H, phenyl–H), 10.87 (s, 1H, NH).^13^C NMR–(δC/ppm): 14.09, 14.43, 55.75, 114.96, 115.40, 115.66, 116.81, 127.44, 127.56, 128.60(2C), 135.02 (2C), 138.0, 144.64, 149.91, 154.77, 165.93, 192.89 (C═N), Analy. Calcud. for Formula: C_19_H_20_N_6_OS (380.14) Elemental Analysis: C, 59.98; H, 5.30; N, 22.09; Found: C, 59.97; H, 5.28; N, 22.06%.

### 3.3 Preparation of Working Electrodes

**FIGURE 11:**
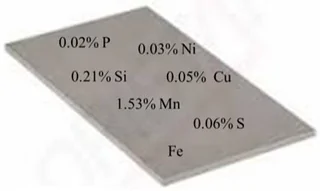
Composition of carbon steel sample.

For corrosion investigations, cylindrical carbon steel (CS) samples containing the following chemical components by weight were used: 0.02% phosphorus, 0.21% silicon, 1.53% manganese, 0.167% carbon, 0.03% nickel, 0.05% copper, and 0.06% sulfur, with iron making up the remaining portion (Figure 11). Emery papers of various thicknesses, ranging from 400 to 2000, were used to polish the metal specimens. The surfaces were polished, then cleaned with distilled water and let to air dry at room temperature. The corrosive solution was made by diluting concentrated hydrochloric acid (37% HCl) with distilled water to create a 1 M HCl medium. Inhibitor solutions with concentrations ranging from 7 × 10^−4 ^M to 5 × 10^−3 ^M were made using the stock solution. Experiments on corrosion were conducted in 1 M HCl with and without different amounts of produced chemicals. Without further purification, analytical‐grade compounds were dissolved in distilled water to create each solution. Every test was conducted using a newly produced solution. A thermostatic control system was used to maintain a consistent temperature of 298 K for the outside testing.

### 3.4 Weight Loss Measurements (WL)

WL and electrochemical studies were used to evaluate the corrosion performance of the examined thiazoles WL analysis was carried out mild steel substrate submerged in 1 M hydrochloric acid at various temperature intervals (298–318 K) for one hour The dimensions of the CS pieces were 2 cm x 2 cm x 0.2 cm. Emery paper with different grit grades (600–2000) was used to abrade carbon steel coupons. They were then cleaned with distilled water, dried, and weighed (W1). Glass hooks were then used to suspend the coupons in 100 mL beakers. The beakers were filled with corrosive solutions both with and without different medication doses. The coupons were taken out after a predetermined amount of time, cleaned with acetone and distilled water, dried, and weighed (W2). The difference between the original (W1) and final (W2) weights was used to compute weight reduction (ΔW). The initial (W1) and final (W2) weights”. The inhibition efficiency (% IE) and surface coverage area (*θ*) were determined using Equation (16) [59]:

(16) $$IE\%=\theta \;\times \;100=[1-\left(\Delta W/\Delta W^{\circ }\right)]\;\times \;100$$

where, Δ*W°* and Δ*W* are the WL in presence and absence of various doses of the ESD. The corrosion rate equation is given by Equation (17):

(17) $$K_{corr}=\left(\Delta W/A\right)/\left(\rho t\right)$$

where: Δ*W *= weight loss (mg), *A* = surface area of the specimen (cm^2^), *ρ* = density of the metal (g/cm^3^), *t* = time of exposure (min) this equation calculates the corrosion rate based on the weight loss of a metal specimen over a given time period.

### 3.5 Electrochemical Measurements

Electrochemical measurements were carried out using an Origamaster 5.0 potentiostat/galvanostat connected to a conventional three‐electrode system placed in a Pyrex glass cell. The working electrode was a carbon steel (CS) specimen with an exposed surface area of 0.384 cm^2^, while platinum served as the counter electrode and Ag/AgCl as the reference electrode. The electrochemical behavior of CS in the absence of prepared compounds was first examined at 298 K, with the system controlled by Origalys software (Version 2.4.0.7). Open circuit potential (OCP) was monitored for 30 min before measurements. Electrochemical impedance spectroscopy (EIS) was conducted within the frequency range of 100 kHz to 0.1 Hz, and Tafel polarization curves were recorded over a potential window of ±300 mV relative to OCP, using a scan rate of 2 mV/s.

### 3.6 The Corresponding Inhibition Efficiency

Characterization of the chemical structures of **3, 5a, 5b,** and **5c** inhibitors; evolution of hydrogen volume over time; polarization plots; mean and standard deviation values for corrosion current density; Nyquist plots; and Bode plots for carbon steel in 1 M HCl with and without different inhibitor concentrations, Table 9.

**TABLE 9:** Potentio‐dynamic polarization parameters of carbon steel (CS) in 1 M HCl containing different concentrations of **3,5a, 5b,** and **5c** along with the corresponding inhibition efficiency (Mean ± SD, *n* = 3).

Inhibitor	Conc. (M)	−E_corr_ (mV)		i_corr_ (µA cm^−2^)		IE (%)		CR (mpy)	
		Mean	±SD	Mean	±SD	Mean	±SD	Mean	±SD
	***Blank***	541	—	0.7182	—	—	—	8.23	—
**3**	7 × 10^−4^	542	±19.0	0.3125	±0.0125	69.58	±1.74	6.29	±0.252
	1 × 10^−3^	494	±17.3	0.2273	±0.0091	74.71	±1.87	3.85	±0.154
	2 × 10^−3^	526	±18.4	0.1701	±0.0068	79.55	±1.99	2.11	±0.084
	4 × 10^−3^	512	±17.9	0.0818	±0.0033	80.12	±2.00	1.08	±0.043
	5 × 10^−3^	509	±17.8	0.0317	±0.0013	82.99	±2.07	0.82	±0.033
**5a**	7 × 10^−4^	555	±19.4	0.2599	±0.0104	58.15	±1.45	4.99	±0.200
	1 × 10^−3^	564	±19.7	0.0992	±0.0040	63.31	±1.58	2.15	±0.086
	2 × 10^−3^	547	±19.1	0.0791	±0.0032	75.83	±1.90	1.35	±0.054
	4 × 10^−3^	451	±15.8	0.0588	±0.0024	82.21	±2.06	0.88	±0.035
	5 × 10^−3^	458	±16.0	0.0233	±0.0009	88.89	±2.22	0.52	±0.021
**5b**	7 × 10^−4^	569	±19.9	0.3298	±0.0132	48.08	±1.20	2.89	±0.116
	1 × 10^−3^	555	±19.4	0.1132	±0.0045	71.23	±1.78	1.75	±0.070
	2 × 10^−3^	576	±20.2	0.0811	±0.0032	82.83	±2.07	0.96	±0.038
	4 × 10^−3^	583	±20.4	0.0458	±0.0018	89.45	±2.24	0.64	±0.026
	5 × 10^−3^	562	±19.7	0.0183	±0.0007	93.89	±2.35	0.42	±0.017
**5c**	7 × 10^−4^	592	±20.7	0.3399	±0.0136	61.28	±1.53	2.59	±0.104
	1 × 10^−3^	584	±20.4	0.1273	±0.0051	79.83	±2.00	1.35	±0.054
	2 × 10^−3^	566	±19.8	0.0701	±0.0028	85.83	±2.15	1.00	±0.040
	4 × 10^−3^	559	±19.6	0.0414	±0.0017	89.20	±2.23	0.78	±0.031
	5 × 10^−3^	568	±19.9	0.0103	±0.0004	94.99	±2.37	0.32	±0.013

Abbreviations: SD, Standard deviation estimated from triplicate independent measurements. E_corr_, corrosion potential. i_corr_, corrosion current density. IE, inhibition efficiency. CR, corrosion rate.

### 3.7 SEM Observation

The surface morphology of copper treated with a concentration of 5 x 10^−4^ of synthetic inhibitors **3, 5a, 5b,** and **5c,** as well as without inhibitors for 6 h, was examined using the ZEISS‐EVO‐UK scanning electron microscope. In order to examine the SEM observation, the surface sheet seems to look into the surface morphology.

### 3.8 Monte Carlo (MC) Stimulation

Using the adsorption locator module included into Material Studio V.7.0 (Biovia/Accelrys Inc., USA), Monte Carlo stimulations of the protonated forms of inhibitors in aqueous solution were carried out [60]. The simulation system was built by cleaving a pure BCC iron crystal with a fractional thickness of 4.0 along the (110) surface plane [61]. The final simulation box size was [24.82 A° x 24.82 A°] [62] after a four‐layers thick iron slab was created using the layer architecture tool and enlarged into a (10 x 10) supercell in order to replicate a strong bulk substrate. To remove unphysical periodic interactions between the surface and the upper boundary, a 50.0 A° vacuum gap was introduced along the *C*‐axis. One copy of the synthetic inhibitor molecule and one hundred explicit water molecules serving as the solvent phase were added to explicitly imitate the corrosion inhibition environment. A target atom set defined by the uppermost surface layer of the Fe (1 1 0) slab with a maximum adsorption clearance distance of 25.0 A° was used to restrict the adsorption search space region in order to suitably confine the configurations. The COMPASS II force field was used for energy evaluations and atomic charge assignments [63]. The Ewald summation approach was used to calculate electrostatic interactions, and an atom‐based cutoff method was used to handle van der Waals interactions. To find the inhibitors' most stable, lowest‐energy adsorption orientations on the iron surface, fine‐quality configurations of simulated annealing were used.

## 4 Conclusion

In this work, new thiazide derivatives were synthesized and characterized using ^1^H, ^13^C NMR, FTIR, and spectral analysis. The electrochemical tests (OCP, potentio‐dynamic polarization, and EIS) are used to assess the inhibitory properties for **3, 5a, 5b,** and **5c**. The inhibitors under investigation showed an increase in inhibitory efficiency (IE%) in the following order: **5c** > **5b** > **5a > 3**. The results show that Compound **5c** is a better inhibitor in 1 M HCl environment than (**5a, 5b,** and **3**), because Compound **5c** has amine and acetyl group. According to the Temkin model, their corrosion inhibition mechanism involves adsorption; the mode of adsorption is classified as mixed type based on the free energy change values. This implies that the distribution of these organic inhibitors on the CS alloy surface in the hostile acidic media is controlled by physicochemical adsorption. At the B3LYP level of theory, quantum chemical calculations based on DFT research were successfully applied to produce ideal molecule structures with electronic computations. The thiazole derivatives **3, 5a, 5b,** and **5c** under examination may have been less stable and more reactive because the estimated global hardness values were lower than the predicted global softness values. The acquired results unequivocally demonstrate that the presence of both an electron‐rich and a π‐electron moiety should affect an inhibitor molecule's electronic structure and make it easier for it to remove water molecules from the metal surface. The theoretical DFT calculations, which offered a clear explanation of the connection between inhibitor molecular structures and inhibition efficacy, were consistent with all experimental results. Using the SEM technique, the development of a strong protective film on the surface of carbon steel was verified.

## Author Contributions

**Ahmed M. Mahmoud,** and **Ghada E. Abd El Ghani** synthesized the compounds, performed the chemical characterization (resources, investigation, and validation). **Ghada Y. Elewady and Abdelfattah M Ouf** wrote part of the original manuscript (visualization, review, editing, and data curation. **Ahmed E. Suliman** performed DFT (visualization, writing, original draft, review, and editing). All authors analyzed and discussed the results and reviewed the manuscript.

## Funding

This research received no external funding.

## Consent

The authors have nothing to report.

## Conflicts of Interest

The authors declare no conflicts of interest.

## Institutional Review Board Statement

Not applicable.

## Supporting information




**Supporting File 1**: cbdv71633‐sup‐0001‐SuppMat.docx

## Data Availability

The data that supports the findings of this study are available in the supplementary material of this article.
