# Supplementary material for: Synthesis, Characterization, and DFT of Some Thiazole Derivatives as Carbon Steel Corrosion Inhibitors in 1 M HCl
Source: Chem Biodivers. 2026 Sep 10;23(9):e71633. doi: 10.1002/cbdv.71633 (PMC13562831; doi:10.1002/cbdv.71633)
Supplement: Supplementary file 1 [file CBDV-23-e71633-s001.docx]

**Synthesis, Characterization and DFT of Some Thiazole Derivatives as Carbon Steel Corrosion Inhibitors in 1M HCl**

Ahmed M. Othman, ^a^ Ghada Y. Elewady, ^a^ Ahmed.E.Suliman^b^, Abdelfattah M Ouf ^a^, Ghada E. Abdel-Ghani^a*^

^a^ Department of Chemistry, Faculty of Science, Mansoura University, Mansoura, Egypt;

^b^ Chemistry Department, Faculty of Science, Menoufia University, Shibin El Kom, Menouia, Egypt

*Corresponding author E-mail: [ghadaemadalden@mans.edu.eg](mailto:ghadaemadalden@mans.edu.eg)

**Figure (S1):** **IR Spectrum of compound 3**

**Figure (S2):** **^1^HNMR Spectrum of compound 3**

**Figure (S3):** **IR Spectrum of compound 5a**

**Figure (S4):** **^1^HNMR Spectrum of compound 5a**

**Figure (S5):** **^13^CNMR Spectrum of compound 5a**

**Figure (S6):** **IR Spectrum of compound 5b**

**Figure (S7):** **^1^HNMR Spectrum of compound 5b**

**Figure (S8):** **IR Spectrum of compound 5c**

**Figure (S9):** **^1^HNMR Spectrum of compound 5c**

**Figure (S10):** **^13^CNMR Spectrum of compound 5c**
